# Iron: The Forgotten Driver of Nitrous Oxide Production in Agricultural Soil

**DOI:** 10.1371/journal.pone.0060146

**Published:** 2013-03-29

**Authors:** Xia Zhu, Lucas C. R. Silva, Timothy A. Doane, William R. Horwath

**Affiliations:** 1 Chengdu Institute of Biology, Chinese Academy of Sciences, Chengdu, China; 2 University of Chinese Academy of Sciences, Beijing, China; 3 Department of Land, Air, and Water Resources, University of California Davis, Davis, California, United States of America; DOE Pacific Northwest National Laboratory, United States of America

## Abstract

In response to rising interest over the years, many experiments and several models have been devised to understand emission of nitrous oxide (N_2_O) from agricultural soils. Notably absent from almost all of this discussion is iron, even though its role in both chemical and biochemical reactions that generate N_2_O was recognized well before research on N_2_O emission began to accelerate. We revisited iron by exploring its importance alongside other soil properties commonly believed to control N_2_O production in agricultural systems. A set of soils from California's main agricultural regions was used to observe N_2_O emission under conditions representative of typical field scenarios. Results of multivariate analysis showed that in five of the twelve different conditions studied, iron ranked higher than any other intrinsic soil property in explaining observed emissions across soils. Upcoming studies stand to gain valuable information by considering iron among the drivers of N_2_O emission, expanding the current framework to include coupling between biotic and abiotic reactions.

## Introduction

Emission of N_2_O from soils is an extensively studied environmental process, given that N_2_O is “at the heart of debates” [1] on several prevalent current issues. Approximately two-thirds of total global emission comes from soils; most of the emission from soils is in turn attributed to agriculture [2]. The intrinsic soil properties (as opposed to temporary changes) most commonly mentioned in research studies and models as controlling emission of N_2_O are texture, pH, organic matter, and ability to supply inorganic nitrogen [3]–[12]. Production of N_2_O in soil is generally attributed to microbiological processes [1], [2], [13]–[17], and therefore the factors that regulate the activity of N_2_O-producing microorganisms should be the same factors that regulate N_2_O production. These controlling factors are generally thought to be well recognized, but as research and related commentary on N_2_O emission from agricultural soils continue to accumulate, the possible role of iron is rarely considered. This is in spite of its known involvement in enzymatic reactions [2], [18], [19] and non-enzymatic reactions [20]–[23] that generate N_2_O. The connection between iron and N_2_O may have been neglected because iron has never figured prominently in routine evaluations of soil for agronomic research or practical management decisions. Unlike the other soil properties cited above, iron does not have a direct and immediate bearing on the growth of most crops or on the agricultural suitability of a soil from either a physical or a chemical point of view. When it is considered, this is in instances of suspected plant deficiency or toxicity, not in the context of its potential connection with the nitrogen cycle. In addition, compared to other intrinsic properties, soil iron does not dramatically affect the short-term changes in microbiological activity generally associated with N_2_O production. For these reasons, once interest in N_2_O began to intensify, the previously reported connection with iron was already out of sight. The intent of our work was to reconsider the potential significance of iron in emission of N_2_O from agricultural soils.

## Materials and Methods

### Ethics statement

The soils used in this study were collected under consent of the land owners, and the compost used was collected under consent of the compost facility management.

### Soil characterization

Soils were collected from the top 15 cm in 10 agricultural fields throughout California, and were sieved to 2 mm following collection. Soil pH was measured in 1 M KCl (1:1 w:v). Percent clay, silt, and sand were determined by a modified pipet method [24]. Total carbon and nitrogen were determined on ball-milled samples by combustion-GC (Costech ECS 4010). Just prior to setting up the experiment, inorganic nitrogen (ammonium plus nitrate) was extracted by 0.5 M K_2_SO_4_ and determined colorimetrically [25], [26]. Dissolved organic carbon (DOC) was determined in the same extract by UV-persulfate digestion (Teledyne-Tekmar Phoenix 8000).

We chose two commonly used, contrasting indices to characterize soil iron: that extractable by acid hydroxylamine (FeA), an index of reactive iron(III) minerals [27]; and that extractable by pyrophosphate (FeP), representing iron complexed with soil organic matter [28]–[30]. FeA was extracted by shaking 0.8 g soil for one hour with 40 ml 0.25 M hydroxylamine hydrochloride in 0.25 M HCl, followed by centrifugation for 30 minutes at 15600× G. FeP was extracted by shaking 1 g soil with 100 ml 0.1 M tetrasodium pyrophosphate for 16 hours, followed by centrifugation for 30 minutes at 15600× G; further centrifugation did not result in any difference in measured iron concentration, indicating that all fine iron colloids had been removed, an important consideration when using this extractant [29], [30]. The concentration of iron in all extracts was determined colorimetrically [31]; pyrophosphate extracts were neutralized by a small addition of HCl prior to this determination. There was no interference from pyrophosphate in the colorimetric analysis. All analyses of soil properties were performed in duplicate. These properties are reported in Table 1.

**Table 1:** Characterization of the soils used in this study.

Location	Classification^a^	FeA^b^	FeP^c^	DOC^d^	Inorganic N	Total N	Total C	Sand	Silt	Clay	pH
		mg kg^−1^	mg kg^−1^	mg kg^−1^	mg kg^−1^	%	%	%	%	%	
*Sacramento Valley*											
Davis	Fine, montmorillonitic, thermic Mollic Haploxeralf	1800	170	17	2	0.09	0.85	30	42	24	5.4
Dixon 1	Fine-silty, mixed, nonacid, thermic Typic Xerorthent	2150	290	30	11	0.14	1.60	23	49	28	5.6
Dixon 2	Fine-silty, mixed, nonacid, thermic Typic Xerorthent	1900	210	19	5	0.11	1.18	15	41	44	5.5
*Salinas Valley*											
Castroville	Fine, montmorillonitic, thermic Ultic Palexerol	710	550	88	32	0.08	0.75	72	15	13	6.4
Salinas 1	Fine, montmorillonitic, thermic Pachic Argixeroll	390	150	44	5	0.07	0.66	64	23	13	7.2
Salinas 2	Fine, montmorillonitic, thermic Typic Pelloxerert	1890	240	88	28	0.16	1.78	22	36	42	7.4
Spence	Fine-loamy, mixed, thermic, Typic Argixeroll	670	270	63	18	0.11	1.28	50	29	21	6.6
*San Joaquin Valley*											
Five Points	Fine-loamy, mixed, superactive, thermic Typic Haplocambid	850	60	57	4	0.08	0.67	36	32	32	6.8
Modesto	Fine-loamy, mixed, superactive, thermic Typic Argixeroll	410	240	164	130	0.11	0.97	72	18	10	6.9
Sanger	Coarse-loamy, mixed, nonacid, thermic Typic Xerorthent	390	260	28	4	0.03	0.30	61	32	7	4.2

a United States Department of Agriculture official soil series description, ^b^ acid hydroxylamine-extractable iron, ^c^ pyrophosphate-extractable iron, ^d^ dissolved organic carbon.

### Experimental treatments

As stated above, the properties most commonly believed to control emission of N_2_O from agricultural soil include texture, pH, organic matter, and the inherent ability of the soil to release inorganic nitrogen. These are intrinsic properties which are not abruptly altered by environmental conditions; in contrast, our treatments were designed to manipulate the most common temporary extrinsic changes that influence N_2_O production: water content, fertilization, and organic amendments. Since these can vary across a range of values, we necessarily limited our choice of treatments. Fertilizer and compost (as a model organic amendment) were either withheld or added at a rate typical of agriculture in California, and two water contents were chosen according to the range expected in agricultural soils. Field capacity, the amount of water a soil can retain against gravity, was chosen as the upper reference point. This is not uncommon, as soil moisture can temporarily exceed field capacity following irrigation or rainfall events [32], [33]. In practice we used water holding capacity (WHC) to represent field capacity. As a contrasting treatment, we chose 50% WHC. This is near the permanent wilting point of most soils [34], and it is not likely that soil moisture will fall below this in the field except during unmanaged dry seasons. Although many intermediate values could have been selected as treatments, we chose to use both ends of a typical spectrum of values in order to present a broad yet concise study.

### Experimental set-up

Prior to set-up, WHC was determined as follows: a soil sample was placed into a funnel lined with filter paper, which was then placed into a beaker of water such that just the tip of the funnel was always in contact with water; after the sample ceased to take up water, the sample was allowed to drain, and the moisture content measured. To begin the experiment, the equivalent of 50 g dry soil was placed into cups, which were themselves placed into larger jars containing a small amount of water to avoid desiccation. The larger jars were sealed with lids containing a small foam plug to allow gas exchange with the atmosphere. To imitate the timing typical of agricultural operations, 2 g finely ground finished green waste compost (corresponding approximately to a field application of 60 t ha^−1^ in the top 15 cm) were mixed with the soils and incubated at 40% WHC for seven days. Treatments not receiving compost were similarly incubated. Following this preincubation, each soil received a fertilizer addition according to treatment: none, ammonium sulfate, or potassium nitrate. The amount of nitrogen added was 100 mg kg^−1^ soil, corresponding approximately to a field rate of 150 kg ha^−1^. Fertilizer solution was sprayed onto the soils to reach a water content of 50% or 100% WHC, depending on the treatment. For each soil there were three replicates per treatment. Samples were incubated for 14 days at 22 degrees C.

Samples for N_2_O analysis were taken on days 0, 1, 2, 3, 5, 9, and 14 following addition of fertilizer. The jars containing the soil cups were closed with lids containing septa and allowed to stand for one hour. Gas samples were taken at 0, 30, and 60 minutes after closure and transferred to evacuated gas sampling vials. N_2_O concentration was determined by gas chromatography-ECD detection (Shimadzu GC-2014). At each sampling date, the rate of N_2_O emission (flux) was determined by linear interpolation of the 0, 30, and 60 minute measurements. Cumulative N_2_O emission over the course of the incubation was calculated using these data, taking the flux measured at a given date to be the average flux for the interval represented by that date.

### Statistical analysis

To identify the soil properties that most strongly explained N_2_O emission in each experimental treatment, we studied the data using partial least squares (PLS) multivariate analysis, a form of structural equation modeling. This tool is particularly suitable when the number of predicting variables is greater than the number of observed variables, when multicolinearity is expected among predicting variables, and when multivariate normality can not be assumed [35]–[37]. PLS ranks the predicting variables by importance based on linear regression models that project the predicting variables and the observed variables to a new, multivariate space. Prior to subjecting the data to PLS analysis, predicting variables (soil properties) and the observed response (cumulative N_2_O emission) were standardized by centering and scaling the data to have a mean of zero and a standard deviation of one. This ensures that the predicting variables are ranked based on how much of the variation is explained when all variables have the same weight.

Although correlations among variables are possible, especially in studies that involve soil properties, this does not change the interpretation given by PLS, which depicts the relative importance of each variable separately, independently of intrinsic links between variables. Nevertheless, a correlation matrix is presented (Table 2) as an aid in understanding the relationships between the soil properties used in our study.

**Table 2:** Correlation matrix of the soil properties evaluated in this study.

	FeA^a^	FeP^b^	DOC^c^	Inorganic N	Total N	Total C	Sand	Silt	Clay	pH
FeA	–	−0.07	−0.41	−0.37	0.68	0.70	−0.91	0.84	0.79	−0.15
FeP	−0.07	–	0.25	0.05	0.04	0.08	0.38	−0.37	−0.31	−0.10
DOC	−0.41	0.25	–	0.93	0.25	0.12	0.53	−0.68	−0.29	0.59
Inorganic N	−0.37	0.05	0.93	–	0.18	0.02	0.45	−0.54	−0.28	0.43
Total N	0.68	0.04	0.25	0.18	–	0.98	−0.57	0.37	0.66	0.47
Total C	0.70	0.08	0.12	0.02	0.98	–	−0.61	0.46	0.66	0.36
Sand	−0.91	0.38	0.53	0.45	−0.57	−0.61	–	−0.89	−0.91	0.13
Silt	0.84	−0.37	−0.68	−0.54	0.37	0.46	−0.89	–	0.63	−0.44
Clay	0.79	−0.31	−0.29	−0.28	0.66	0.66	−0.91	0.63	–	0.17
pH	−0.15	−0.10	0.59	0.43	0.47	0.36	0.13	−0.44	0.17	–

a acid hydroxylamine-extractable iron, ^b^ pyrophosphate-extractable iron, ^c^ dissolved organic carbon.

Following the exploratory PLS analysis, linear regressions between iron and N_2_O emission were calculated using unweighted, untransformed data, and were considered significant enough to report at P<0.1. All statistical analyses were performed using JMP 10 software.

## Results and Discussion

**Figure 1:**
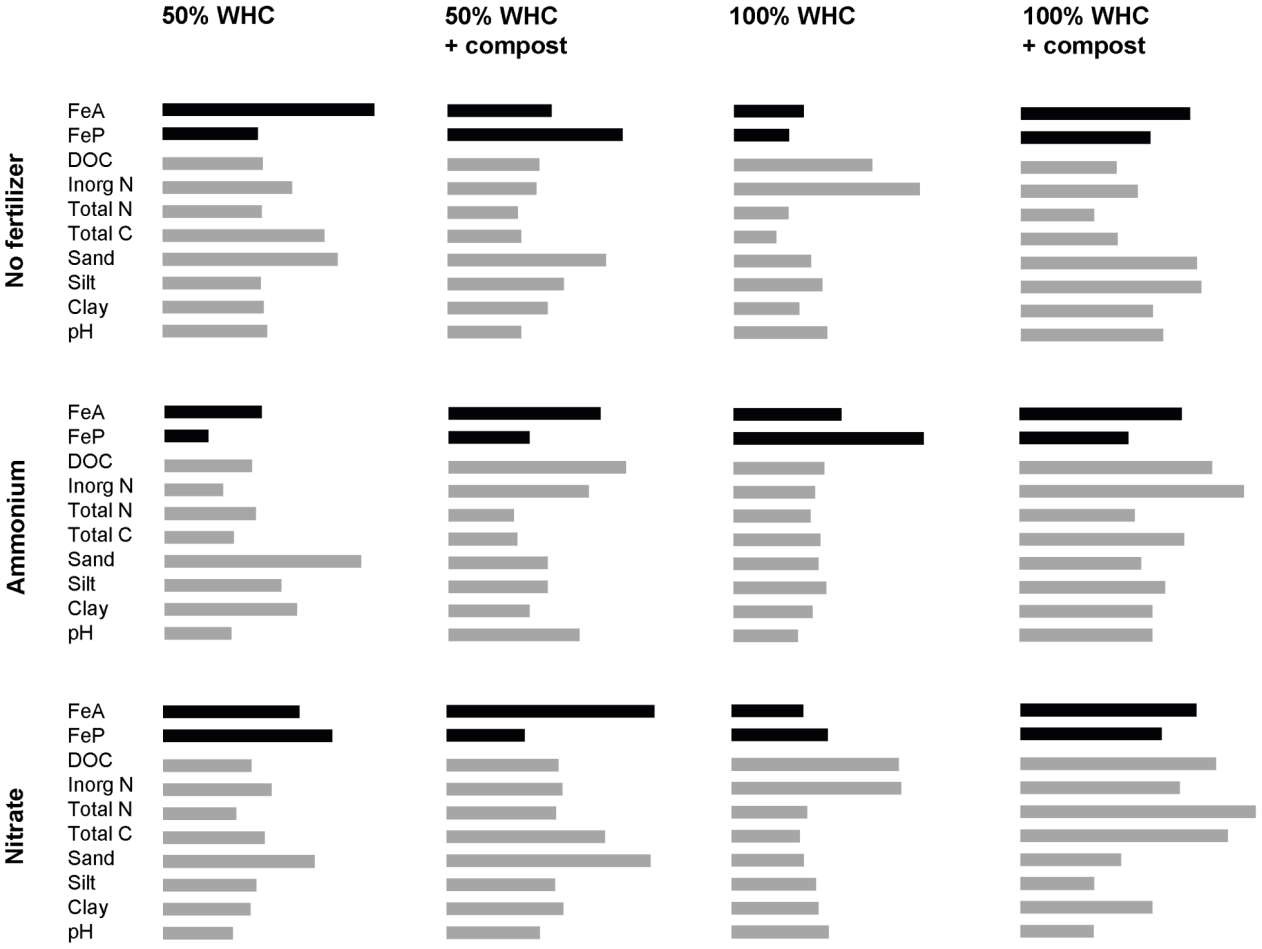
Relative importance of soil properties in explaining cumulative emission of N_2_O under different conditions. Result of partial least squares multivariate analysis performed across ten soils for each of 12 different treatments. Two indices of soil iron (FeA: acid hydroxylamine-extractable iron and FeP: pyrophosphate-extractable iron) were ranked alongside other soil properties commonly considered to control soil N_2_O emission. The size of each bar is given by the variable importance in the projection (VIP) value, and indicates the relative strength of each variable in explaining emission in that treatment. WHC =  water holding capacity; DOC =  dissolved organic carbon.

The results of the PLS analysis are shown in Figure 1, where each soil property is ranked according to its ability to explain cumulative N_2_O emission across all soils. This ranking was performed for each of the 12 different treatments studied. In five of these treatments, iron (as either FeA or FeP) ranked higher than any other measured soil characteristic in explaining observed emissions. In four additional treatments, iron was among the top four predictors.

As a complementary approach to further investigate the relationship between iron and N_2_O emission, simple linear regressions were calculated in which N_2_O data were compared against FeP and FeA. Whereas PLS was used to arrange a suite of soil properties according to their ability to explain N_2_O emission, regressions indicate, by the value of r^2^, how much of the variability in N_2_O emission can be explained by a single property; regressions also indicate the direction of the effect (positive or negative slope) and degree of importance of the effect (absolute value of the slope). In most cases, a significant relationship between N_2_O emission and a given variable can be expected when that variable is ranked highly by PLS. In certain cases, however, a variable ranked highly by PLS may not necessarily yield a significant linear relationship when that variable is considered apart from the other variables; conversely, certain treatments in which a variable is not ranked highly by PLS may nonetheless yield a significant regression. The primary reason for this occasional discrepancy is the nature of the PLS procedure: by considering all predicting variables together, new predictors are generated which are composites of the original variables. Table 3 reports the results of the regressions for treatments that showed a significant relationship between N_2_O emission and either iron index. Despite a dataset of values for N_2_O emission which spanned more than three orders of magnitude across soils, several notable connections between iron and N_2_O emission emerged.

**Table 3:** Results of simple linear regression of cumulative N_2_O emission (as ng N_2_O-N g^−1^ soil) against iron, across ten soils and under 12 different conditions.

	50% WHC	50% WHC + compost	100% WHC	100% WHC + compost
No fertilizer	NS	FeP: 0.37, 0.38	FeA: 0.12, −0.09	NS
Ammonium	NS	FeA: 0.28, −0.20	FeP: 0.62, 11.9	FeA: 0.23, −0.62
Nitrate	FeP: 0.19, 0.46	NS	FeP: 0.16, 2.1	NS

The first value given is that of r^2^, and the second value is the slope of the regression. NS =  regression was not significant for either iron index. WHC =  water holding capacity; FeA =  acid hydroxylamine-extractable iron; FeP =  pyrophosphate-extractable iron.

FeP was significantly related to N_2_O emission in four treatments, in which it explained between 16 and 62 percent of the variability, with a positive slope in all cases (i.e. greater emission was associated with more FeP). This influence was greatest under 100% WHC when ammonium was present and compost was absent. Such a condition may be reasonably expected on occasion, since most fertilizers supply ammonium, and since this may occur close in time to irrigation or rainfall. In this treatment, an increase in FeP of 1 mg kg^-1^ corresponded to an increase in cumulative emission of 11.9 ng N_2_O-N g^−1^ soil (averaged across all soils) during the course of the incubation (Table 3).

Like FeP, the connection between FeA and N_2_O emission was also significant under several different conditions. Unlike FeP, however, which was positively related to N_2_O emission, FeA was always negatively related to N_2_O emission. There was no treatment in which both iron indices were significantly related to N_2_O emission (Table 3). Considering that FeP and FeA bear almost no relationship to each other (Table 2), this difference in behavior suggests that these two indices indeed reflect two forms of iron that differ in reactivity. Also notable in Table 3 is the effect of compost in fertilized treatments: the observed negative association between N_2_O emission and FeA occurred only in the presence of compost, while the stimulating effect of FeP was observed only without compost.

The contrasting relationships of FeA and FeP with N_2_O emission could be due to differences in the reaction of either form of iron with nitrogen compounds in the soil matrix. For example, hydroxylamine is produced from biological oxidation of ammonia, and is known to generate N_2_O upon chemical reaction with iron(III) [20], [38]. Reaction with FeP versus FeA, or locally high concentrations of either hydroxylamine or iron, could lead to more or less N_2_O compared to other reaction products [38]. The ability of aerobic microorganisms to acquire iron can likewise depend on its chemical nature, consequently influencing the amount of reactive compounds produced or consumed through reactions that use iron-dependent enzymes. As soil water content increases, reducing conditions may develop, especially when the depletion of oxygen is accelerated by easily metabolized organic matter. The chemical nature of existing iron(III) may determine the ease with which it is reduced to iron(II) in anaerobic microsites. This will in turn control its participation in other reactions that produce N_2_O, such as chemodenitrification, which includes the abiotic reduction of nitrite to N_2_O by iron(II) [39], [40]. Chemodenitrification can also produce other gases, and the relative amount of N_2_O released may be affected by the form of iron present. A related anaerobic process is nitrate-dependent iron(II) oxidation [41]; a recent review [42] has highlighted, in the context of this process, how the simultaneous presence of nitrate-reducing and iron(III)-reducing areas can potentially be important to nitrogen cycling. Under anaerobic conditions, iron(III) can also be linked to ammonium oxidation [43], [44]. If reactions that generate N_2_O are active in any of the above processes, they may be stimulated or suppressed by different forms of iron, such as the two indices examined in this study. The degree of this influence under different conditions will then determine the importance of iron relative to other soil properties.

Our treatments consisted of two contrasting values for soil moisture and addition of amendments. This was done in order to explore the importance of iron across a wide range of conditions while at the same time avoiding a cumbersome dataset. It is clear from Figure 1 that the importance of iron can change between the two limits of each treatment variable. For example, between 50 and 100% WHC under ammonium fertilization, iron moves from a position of modest relevance to become the highest-ranked driver. Since our results show the importance of iron only at two distinct values, we do not know how its importance under intermediate conditions changes between the two end values. Even without such intermediate data, the differences between contrasting treatments can aid in understanding the mechanisms at work in generating N_2_O. In the above example, the importance of iron rises markedly under ammonium fertilization as soil moisture increases from 50 to 100% WHC; FeP surpasses FeA in strength as well. As mentioned earlier, ammonia is oxidized to hydroxylamine, and this can react with iron(III) to produce N_2_O. In a wetter soil, solutes are more mobile, which can lead to greater production of hydroxylamine as well as greater contact of hydroxylamine with iron. FeP is also likely to be more soluble than FeA. Any combination of these effects might elevate the importance of iron and change which form is more relevant in explaining the associated N_2_O data.

The overall position of iron among other drivers of N_2_O emission is determined by both its reactivity and the presence of processes subject to its influence. Ample opportunity for inquiry exists for defining the extent of the relationship between iron and N_2_O in managed as well as unmanaged ecosystems, and this can provide useful practical and theoretical information. For example, including iron in current models of N_2_O emission may strengthen their predictive ability. In addition, inasmuch as certain indices of iron can be related to its physical or chemical characteristics, observing the relationship between a given index and N_2_O production, and how this changes under different conditions, may provide insight into the specific reactions at work. As stated earlier, production of N_2_O is generally accepted to be a microbial affair, and it is logical to assume that the factors that regulate the activity of N_2_O-producing microorganisms should be the same factors that regulate N_2_O production. This is not incorrect, but is perhaps a somewhat restrictive rendering; a more accurate framework might include “biotic-abiotic reaction sequences” [39] that generate N_2_O, such as those outlined above. Indeed, “the complex interactions that occur between microorganisms and other biotic and abiotic factors” have been suggested to be a key part of further understanding greenhouse gas production and improving predictions [17].

## Conclusion

It has been recently emphasized [45] that solutions to environmental problems require explicit consideration of the couplings between element cycles. The environmental chemistry of iron has been well researched, as have many of the interrelated details of the nitrogen cycle. The specific connection between iron and N_2_O in soil has also been recognized in both older and recent studies. However, iron and nitrogen have yet to be brought together in agricultural systems, the foremost source of soil N_2_O emission. Our most important conclusion is simple: iron does indeed figure prominently among the soil properties controlling N_2_O emission in contrasting conditions across diverse soils. Studies concerned with the potential of agricultural soil to emit N_2_O will gain new momentum by remembering this “key biogeochemical engine” [46], building on a connection identified a long time ago but largely overlooked since then.

## Acknowledgments

We gratefully acknowledge the assistance of those who collected the soil samples, as well as the constructive comments received during review, which were very helpful in improving our paper.
